# Mental healthcare-seeking behavior during the perinatal period among women in rural Bangladesh

**DOI:** 10.1186/s12913-022-07678-z

**Published:** 2022-03-07

**Authors:** Goutam Kumar Dutta, Bidhan Krishna Sarker, Helal Uddin Ahmed, Dipika Shankar Bhattacharyya, Md. Musfikur Rahman, Ratna Majumder, Taposh Kumar Biswas

**Affiliations:** 1 icddr,b, 68 Shaheed Tajuddin Ahmed Sarani, Mohakhali, Dhaka, 1212 Bangladesh; 2 National Institute of Mental Health and Hospital, Dhaka, Bangladesh; 3 Maternal and Child Health Training Institute, 1205 Dhaka, Bangladesh

**Keywords:** Healthcare-seeking behavior, Maternal mental health, Community level in Bangladesh

## Abstract

**Introduction:**

Mental health conditions are of rising concern due to their increased contribution to the global burden of disease. Mental health issues are inextricably linked with other socio-cultural and health dimensions, especially in the rural areas in developing countries. The complex relationship between mental health issues and socio-cultural settings may largely toll upon healthcare-seeking behavior. So, it urges to document the current status of mental healthcare-seeking behavior during the perinatal period among rural women in Bangladesh to develop a context-specific intervention in the future.

**Methods:**

This study was carried out in one sub-district in Bangladesh from April 2017 to June 2018. We conducted 21 In-depth Interviews (IDIs) and seven Focus Group Discussions (FGDs) with different groups of purposively selected participants. After collecting the recorded interview and making the verbatim transcription, the data were coded through *Atlasti* 5.7.a. Data were analyzed thematically to interpret the findings.

**Results:**

Two-thirds of the total respondents did not seek mental healthcare during the perinatal period at the community level. They also did not know about the mental health service provider or the facility to get set these services. Only one respondent out of twenty-one sought maternal mental healthcare from a gynecologist from a private hospital. Socio-cultural factors such as social stigma, traditional beliefs and practices, social and religious taboos, and social capital negatively influence healthcare-seeking behaviors. Besides, the community-level service providers were not found to be adequately trained and did not have proper guidelines regarding its management.

**Conclusion:**

The findings provide evidence that there is an urgent need to increase the awareness for service users and formulate a guideline for the community-level service provider to manage maternal mental problems during the perinatal period of women in rural Bangladesh.

## Introduction

Globally, mental health conditions are of rising concern due to increased contribution to disease burden [1]. Mental well-being is inextricably linked with the social and physical environment. Thus it, cannot be determined by only the absence of mental disorders but also by related socio-economic, biological, and environmental factors [2]. Mental health disorders refer to a set of medical conditions that can affect a person’s thinking, feelings, mood, ability to relate to others, and daily functioning [3]. Poor maternal mental illnesses affect more than 1 in 10 women during pregnancy and the first year after childbirth and can devastating impact on them and women [4–6].

Due to physiological changes during the perinatal period -defined as the time spans from conception to when the infant reaches the age of one, many women are affected by mental disorders [5]. However, there is still a big concern over how pregnant mothers seek care for mental health problems, especially in low and middle-income countries [7]. Evidence suggests, only 13.6% of women have sought help for their depressive symptoms [8]. Healthcare-seeking behavior and the socio-cultural influencing factors have a considerable impact on the lives of women as well as on the early childhood development (ECD) of the baby [4, 9]. In terms of the perinatal health services in low and middle-income countries where women’s familial, social, and physical environment are crucial determining factors, the decision to seek care is highly complicated [2, 9–13]. Besides, at the population level, there is also a lack of clear understanding among the service users regarding where to seek service for particular types of disease, which often hampers appropriate and timely care-seeking [14–18].

Mental health issues have been incorporated as an essential prerequisite for good health and well-being in the United Nations (UN)- declared Sustainable Development Goals (SDGs) [19]. Therefore, mental health support services are needed to ensure the perinatal mother’s good health and well-being, at the community level. But there is a considerable knowledge gap regarding the availability and accessibility of these services in Bangladesh, especially when there are traditional beliefs and social taboos [20] which ultimately hinder them from seeking care for mental health problems fearing discrimination [14].

Understanding healthcare-seeking behavior in a community is necessary to develop appropriate health policies, health systems, and educational strategies to facilitate access [10]. Besides, its determinants of optimal healthcare-seeking behavior of perinatal women in this period can significantly reduce the impact of severe illness on children’s growth and development [21]. Given the variability of socio-demographic and cultural contexts, there are differentials in the perception of vulnerability or risk for newborns and prevailing customs, traditions, and beliefs within communities. Therefore, it is critically important to understand community-specific patterns and determinants of population-level antenatal, delivery, and postnatal care-seeking practices, especially for the perinatal period of women. The public health system in Bangladesh is well-organized, starting from the community level to the national level. Still, there is a lack of research-based data or evidence for estimation, service, or support for a particular group of people, service provider, and budget (0.05% is designated for mental health services) to promote perinatal mental healthcare of women in community settings. Since there is a lack of enough evidence around community people’s knowledge and health service providers’ expertise on psychosocial support to perinatal women in rural Bangladesh. From this context, this paper aims to explore the healthcare-seeking behavior and the influencing factors for healthcare-seeking and document the service gaps at the community level for perinatal mental disorders of women in Bangladesh.

## Research design and methods

It was formative cross-sectional research where we applied a qualitative approach for data collection [22]. We employed thematic analysis for this study to describe the meaning and significance of respondents’ experiences regarding healthcare-seeking behavior of women’s perinatal mental health. We conducted both IDIs and FGDs to collect data from different populations.

### Study settings

The study was carried out at the community level in one sub-district in the *Rajbari* district of Bangladesh. We purposively selected the study area based on the low proportion of care-seeking during the perinatal period and the high mortality rate of under-five children [23]. The *Upazila* (Local administration in sub-district level) is divided into one municipality and seven unions (the smallest administrative unit in Bangladesh). We covered all unions of this *Upazila* for the study.

### Study population, sample size, and sampling criteria

We carried out 21 IDIs in this study with a different group of people. Among them, 14 IDIs were conducted with mothers who had at least one-year-old children to know their perception of having mental health issues and experience of seeking care for these illnesses. Besides, seven IDIs were conducted with the community service providers such as Sub-Assistant Community Medical Officer, Family Welfare Visitor, Family Welfare Assistant, Health Assistant, Community Health Care Provider (CHCP), and Non-Formal Practitioner. We labeled this particular form of data saturation to fix the number of IDIs in this study. Data saturation refers to reaching a point of informational redundancy where additional data collection contributes little or nothing new to the study [24]. In this regard, Cohen et al.,2000, p. 56 suggested that between 10 and 30 interviews are the best to explore an objective in phenomenological research [25]. All the IDI participants were selected purposively considering the types of respondents and willingness to participate in this study as an interviewee [26]. To triangulate data, we also conducted seven FGDs with community stakeholders like household heads, Union Council (A Union Council consists of an elected chairman and twelve members including three members exclusively reserved for women) members, teachers, religious leaders, and service users who took other services without maternal health care. For the selection of FGD participants, we considered heterogeneity regarding age, sex, education, occupation, etc., to gather information for a different aspect of the participants. However, before selection, we did not use any diagnostic tool to identify the mental illness in this study. Therefore, all of the interviewed participants were undiagnosed whether they had any mental illness or not.

### Data collection and quality control

The data collection guidelines were developed separately for different groups. The data collection tools were finalized after incorporating of findings from pre-testing done with a similar group of the population living in another area far from the study site. Then, it was translated from English to Bengali and in the local dialect for conducting interviews due to understanding their local dialect as an outsider. Furthermore, to ensure reliability, we assessed the meaning and explored health and mental healthcare-seeking behaviors in different socio-cultural influencing factors during the perinatal period of women at the community level. Content validity was assured by seeking confirmation of each health belief by other women on different days to understand each from various sources. We ensured comprehensive collection and assessment of the socio-cultural health beliefs and practices on healthcare-seeking behavior during the perinatal period of women in the study area. A data collection team with two Senior Research Assistants and one Research officer, experienced in qualitative data collection, was formed. The data collection team has been trained intensely on all pros and cons of data collection, including consent taking, different data collection methods, antenatal, delivery, postnatal maternal mental health problems of women, and community health service in the perinatal period. The team visited the household of the selected antenatal and postnatal women, and mothers who had recent experience in child birthing and caring at their homes for conducting IDIs. The IDIs were taken one to one basis at the home of the respondents and took 30–40 min each. In addition, seven FGDs were conducted with 7–8 respondents at Community Clinic (CC) and took 40–50 min each. To obtain data, we followed the saturation level of information. Data were checked every day through feedback sessions at the end of the day. We listened to the recorded interviews to identify new issues and find out any missed opportunities to further explore them. A central monitoring team of investigators was involved in continuous monitoring of the data collection to ensure quality.

### Data analysis

All the data were collected through audio recording along with note-taking. The audio recordings were transcribed verbatim (in their original form). Then the transcripts were organized through cross-checking with the interview notes. Transcripts were randomly checked against audio recordings to ensure the quality of the transcription. The data were analyzed using a thematic approach. We identified themes, as per the research objectives. The transcribed data were systematically coded, synthesized, and interpreted to explain the findings. Results on the same issues from different types of respondents and areas were compared to strengthen the validity of the findings. We used *Atlas ti 5.7.a.* software for coding and organizing the data.

## Results

We have categorized four broad themes to explore maternal mental healthcare-seeking behavior of the perinatal women in the study area. In the first theme, we showed the essential characteristics of the participants that can influence their decision during this period. After that, we explored the respondents’ perceptions of maternal mental health problems. Then, we accumulated a broad theme named maternal mental healthcare-seeking during the perinatal period of women. Next, we investigated under this theme into how the respondents recognized perinatal mental health and where we sought perinatal mental healthcare, and who provided this support in seeking care during the perinatal period. Finally, we found out the socio-cultural factors and neighborhood support that influence care-seeking practice.

### Socio-demographic characteristics of the respondents

A total of 74 participants (21 from 21 IDIs and 53 from seven FGDs) participated in the study. The study participants’ socio-demographic characteristics revealed that the highest percentage (64%) of study participant’s age was 30 years or above. Among the total participant’s 35% was male and 66% female. Most of the participants (87%) were Muslims and the rest of the participants (14%) were Hindus by religion. About one-third of study participants (32%) had higher secondary or above level education, 34% had secondary level education, but one-fifth of the participants had no formal education. Among the study participants, 39% were housewives, 39% were service holders, 11% were businessmen, 8% were farmers, and 4% were teachers. There were also members of local governments such as *Upazila Parishad* (local administration in sub-district level), religious leaders, and retired persons. Forty percent of the participants had a monthly household income of more than 12,000 takas, while 24% had fewer than 3000 takas.

### Perception on maternal mental health problems

Two-thirds of the respondents said that maternal mental health problems are not a problem to them during the perinatal period of women because it’s a natural phenomenon. The mood swings, dizziness, bad dreams in the sleep, and the fears of death for pregnancy, that the mother experiences are explained due to the extra burden of being pregnant and child-rearing this period and are viewed as usual symptoms of this period. Therefore, additional support or medications are not deemed necessary. In addition, these symptoms are regarded as usual for women not labeled as a disease condition to treat.

On the other hand, mental health issues are significantly related to the matter of social stigma for a woman in the community. If the woman has mental diseases, they call her “*pagol”* (mentally sick/mad)*.* So, they think that woman has to seek treatment from Mental Hospital locally known as “*Pagla Garod”* (loosely translated as a sanctuary for the mad people). Five respondents said that they were reluctant to seek healthcare due to this perception of maternal mental health and any mental health in the study area. They do not know who serves this support or medication and practitioner in the community (primary level) for mental health, especially maternal mental health problems during the perinatal period of women. One respondent (mother) said that,

“*I felt worried during pregnancy. I think this is typical for women in the perinatal period. But I did not seek any doctor. Even I did not know who provides the treatment.*” (Age: 19 years’ female, Education: class five, Occupation: Housewife)

Two-thirds of community service providers said they did not hear about the women’s perinatal mental health problems. Instead, they listened to their colleague’s mental health diseases names, such as depression, anxiety, stress, etc. They reported that they provide counseling for taking nutritious food, preparing for arranging money, and vehicles for the emergency period. Apart from these, they did not provide any psycho-social counseling during the perinatal period of women for their mental health and well-being. One community healthcare provider said maternal mental health is closely related to their circumstances and social and physical factors during this period. Suppose they cannot treat any symptoms of women. In that case, they can refer to the Upazila Health Complex (UHC), a high-level facility of primary level healthcare in Bangladesh. So, they cannot suggest any support and management for mental health-related problems like depression, anxiety, stress, and postnatal psychotic disorders in general due to the absence of training and treatment guidelines.

### Maternal mental healthcare-seeking during the perinatal period

#### Recognitions of antenatal mental health and care-seeking

All of the respondents said that when women conceived, they locally called her ‘*poati’* (pregnant), *pet hoice* (being pregnant), or ‘*Maa hote cholechhe’* (mother-to-be). After being confirmed, they inform their senior family members (mother-in-law if present, husband). In most cases, the husband and/ or aging family members decide to seek care if needed. The three-fourths of the participants reported that being pregnant is not a serious issue requiring doctors. Regarding maternal mental health problems, two-third of the respondents reported that pregnancy might be associated with would-be mother’s mental concerns. In this connection, many mothers have experienced fear of delivery, pounding heart, and sweating (anxiety) for their upcoming child’s good health and well-being, the impending birth, during the pregnancy period. They also added that poor mental health conditions might lead to increased risk in childbirth, followed by postnatal mental illness and improper child care. Some women had a mental illness when they became pregnant, and some had mental health problems during the maiden pregnancy. One respondent described her experience as,

“*After over my menstruation date, I felt a change in my appetite. I could not eat anything and felt uneasy; I thought about what had happened to my body! Then I took a quick pregnancy test, and the result was positive. So, I was very nervous. My husband told me; he was happy about that. I could not express my mood at that time.”* (Age: 18 years’ female, Education: class one, Occupation: Housewife)

One respondent reported that since the husband is not aware of the mental state of women during pregnancy, the wife does not get any support from them that makes the mental problems worse. A few respondents reported that husbands had seen the adverse mental condition of their wives in the perinatal period regarding pregnancy complications. For example, a member from an FGD said,

“*One day, my wife told me, please forgive me for my any fault if I die while giving birth. My wife felt fear of her delivery and related danger signs. Sometimes, she dreamt like this since I did not recognize that it was mental health problems.*” (Age: 32 years’ male, Education: Higher Secondary School Certificate, Occupation: Businessman)

The other dimension of mental health is closely related to the sex of the upcoming baby. For example, one respondent (Husband) reported that his wife was tense due to her expectation for a boy baby. He quoted,

“*They were agitated during the fourth pregnancy because they already have three daughters. If it is repeated (daughter), what will happen then? This made them anxious.*”

Two-thirds of the respondents (service users) said that there are no mental service providers in the primary and secondary level hospitals in the health system in Bangladesh. Only one respondent out of twenty-one sought maternal mental healthcare from a gynecologist in a private hospital.

From the supply side perspective, at the community level, the service provider (CHCP) mentioned that they do not have any guidelines and knowledge to provide maternal mental disorders in the perinatal period of women. One respondent shared her experiences,

*“When I was pregnant, I felt apprehension or dread, tense about my delivery, and panicked regularly. Then I shared it with my husband. He told me to go to CC for taking counseling, but Apa (CHCP), could not provide any suggestions on these.”*

One-third of the respondents (services providers and stakeholders) opined that unintended pregnancies happened in most cases at the community level in our country, making pregnant women mentally depressed.

#### Delivery care-seeking

In terms of physical health, two-thirds of the respondents reported that they sought treatment during the delivery period from a private hospital, clinic, or Mother & Child Welfare Centre (MCWC), popularly known as “maternity” at the district level. A few respondents also revealed that they also went UHC for delivery purposes during the delivery period. Five out of fourteen respondents informed that they sought delivery care from the district hospital. Among all the respondents, only one respondent went to Faridpur Medical College and Hospital, a tertiary level hospital for delivery care seeking due to prolonged labor pain. In terms of mental health care seeking, two-thirds of the respondents said that they felt tensed and became frustrated over the danger signs, fatigue, hopelessness, body pain, and labor pain during the delivery period. During the delivery period, they did not seek doctors’ treatment for these types of maternal mental crises. They seem that it is a more natural process for human beings and will be cured naturally. Three respondents said that they are followers of *Atrashi pak Darbar Sharif (religious and spiritual place)*; they get talisman (spiritual healer) from this *Darber Sharif* for any mental health problems during the delivery period. Another two respondents opined that they took *pani pora* (blessed water) from the mosque’s Imam (Muslim religious leader) to cure worries during the delivery period.

#### Postnatal (6 weeks or 42 days after delivery) care-seeking

Two-thirds of community-level service providers reported that they do not have formal knowledge and treatment guidelines to deal with postnatal mental disorders such as depression, anxiety, stress, postpartum psychosis, post-partum blue, and related symptoms for the postnatal period of women. Even if they have no idea that poor mental health conditions may lead to increased risk in childbirth followed by postpartum depression. One respondent said,

“*I think maternal mental disorders have seen in the perinatal period, especially after delivery due to her physical poor health conditions and new kid’s crying and disturbance.”* (Age: 18 years female, Education: Secondary School Certificate, Occupation: Housewife)

On the other hand, five respondents said that they have experienced mental health problems during the postnatal period although they did not seek treatment. One mother reported that she thought of seeking mental health treatment during the postnatal period, but she did not know where to go for the treatment. Only one respondent involved in the teaching profession revealed that depression, anxiety, postpartum blue, and psychosis are the most common mental health problems in the postnatal period, but treatment management is not available in the community level facility.

### Socio-cultural influencing factors

In the study area, two-thirds of the respondents revealed that healthcare-seeking behavior had been influenced by many confounding factors furthermore, some factors have more significant influences on maternal mental disorders treatment seeking as follows; socio-cultural and religious beliefs, practices, taboos, and restrictions during the perinatal period of women. These factors have been elaborated by respondents’ experience below.

#### Beliefs and practices

Two-thirds of community people have different beliefs in social and religious entities on maternal health and mental health issues during the perinatal period. Often the traditional healers, religious leaders, folk, and spiritual healers are referred to as the sources for treatment-seeking. Three out of 14 respondents revealed that they did not go to the doctor to seek mental healthcare during the pregnancy period because the doctors might give tests for pregnant women that might be harmful to the fetus. Besides, it was also costly to go to doctors. In that case, they had to abide by their mother-in-law and husband’s decision that led them to go to the religious leaders for spiritual blessings. Three respondents out of 14 said they sought treatment from *Sasto Kormi (Health worker) of* Bangladesh Rural Advancement Committee because they knew her and cared for community people through household visits.

#### Support from neighborhood

Two-thirds of the respondents reported on this common issue that social capital was a leading social determinant to motivate maternal mental healthcare-seeking behavior in the perinatal period of women. Through this relationship, a person gets support to improve the mental well-being of women in the perinatal period. Two-thirds of pregnant women who participated in this study explained that they usually got help from their neighbors during pregnancy and in any critical situation. One pregnant woman quoted,

“*I felt severe pain in the lower abdomen during the eighth month of my pregnancy. I did not find any way. I shared with my husband, but he did not solve the problem from his knowledge; then I went to my neighbors. She told me that it is very usual in the pregnancy. I got relief after hearing this”*. (Age: 28 years’ female, Education: class seven, Occupation: Housewife)

Another female respondent who has 1 year’s child stated,

“*When I conceived, I did not know who would be better for medication at that period. My neighbor told me to go to either the private clinic or Maternity in Rajbari Sadar. After that, I went to maternity and got checked by* a gynecologist.*”* (Age: 18 years’ female, Education: class seven, Occupation: Housewife)

Two-thirds of respondents opined that husbands’ support is the most trusted and closest support than female relatives and friends. So, his support is considered the most important support during pregnancy. Besides, the intimacy in the husband-wife relationship has a central role in social support received and their sense of togetherness.

## Discussion

Our study suggested that healthcare-seeking behavior regarding maternal health and mental health disorders during the perinatal period has a considerable impact on the lives of women during the perinatal period. Moreover, the finding also emphasizes that healthcare-seeking practice is like a process that begins at the community level and ends with the specialized doctor in the sub-district or the district level.

However, the result shows rural women does not usually seek treatment for physical health and mental health problems during the perinatal period. This finding is similar to other studies showing that parents are reluctant to seek mental health issues [27]. Even 78% of parents sought ‘no care’ for their preterm newborn [28]. Our result shows pregnant women faced many problems during perinaltal period like sadness, eating disorder, fear for delivery unable to proper sleep, tension (excessive thinking about the outcome), but they rarely go to seek care for identifying or differentiating to normal sadness or some pathological conditions like anxiety, stress, etc., from pathological depression, anxiety, trauma etc. those lead to mental disorders. Similar finding from a systematic review that, women do not seek medication for any maternal mental health disorders during the perinatal period of women [29].

At the individual level, married pregnant adolescent girls usually avoid health facilities for pregnancy and delivery care because of their perception that pregnancy is a natural phenomenon. Therefore, there is no need to receive pregnancy care and other medical supports. Sometimes, shyness is seen for male service providers, and some women are found to be afraid of instrumental delivery and surgical intervention if needed. In interpersonal and family-level factors, they pointed out that decision-makers (husband, mother-in-law, senior family members, and relatives) play an essential role in using skilled maternal health services. Across the low and middle-income country settings, there is still a big concern over how pregnant mothers seek care for mental health problems, especially in as evidence suggests, only 13.6% of women have sought help for their depressive symptoms [9].

Even our research found that some women went to discuss with the community service providers but did not get any fruitful treatment or effective support on mental health problems during the perinatal period as the service providers are not oriented about the perinatal mental health problems. So, the healthcare providers at the community level need to know the proper guidelines for the initial management of mental health problems and appropriate referrals. A similar finding was reported in another study that a high prevalence of antenatal depression among rural women, who rarely seek treatment for their depression which goes with our study findings [7]. Gausia K. et al. 2009 revealed that 14% of women with depression admitted that they felt like doing self-harm during their current pregnancy [30].

Findings show that most mothers stated that they felt panicked, phobia, stress, physical aches, and pains during the delivery period. Still, there are no people beside them to give mental support. Similarly, other studies found that most respondents seek healthcare from traditional birth attendants (TBA) and Non-Formal Practitioner during the delivery period though they do not have any knowledge or training regarding maternal mental health. In many other pieces of literature that delivery usually takes place at home and is attended by TBA [31, 32]. On the other hand, a national survey in Bangladesh found that the health facility delivery rate is very low [33], similar to this study’s findings. So, they think it is a natural process that is obvious in life. But they felt a need for mental support during the delivery period [34].

Our study found that the religious beliefs and practice of family influence care-seeking. Almost similar findings were found from a study that showed women sought faith-based medication from ‘*local religious leaders* ‘along with formal Healthcare services [10]. Likewise, another study found that women receive pregnancy-related care from multiple sources based on the nature and type of threats they associate with their pregnancy [35]. Moreover, there is an influence of social capital on healthcare-seeking behaviors on maternal health and mental health. From the same aspect, Ahmad R, et al., found a strong influence of healthcare-seeking behaviors during the perinatal period of women. This study found that women’s social networks help seek treatment as needed [36]. In another study by Yakong, V N et al. found that the healthcare-seeking behavior of mothers during the perinatal period was influenced by interpersonal communication between providers and patient parties. That study further showed that women received treatment surreptitiously for mental health-related disease but some patients experience a lack of privacy during treatment-seeking [37].

Besides, women’s help-seeking was influenced by their expectations and experience of healthcare professionals and the healthcare system’s structural factors [9]. We found in this study that community health care service providers had no necessary training and a guideline for the patient’s management who has mental health problems during the perinatal period. That’s why perinatal women cannot get adequate services to meet the requirements regarding mental health problems. Another study also found that inadequate service providers and their management guidelines are the vital barriers to providing services for mentally disordered women during this period and hampers the quality of life of people in developing countries [34]. However, the findings suggest that the community health service providers should be capacitated by providing training on identifying mental disorders and the proper management, including referral. Along with this, different awareness campaigns regarding perinatal diseases at the community level should be arranged by the government and non-government development partners to educate community women regarding the mental disorder and inform them about the appropriate service points.

### This study has several limitations

Firstly, this is a relatively small-scale study with a small sample size that does not represent the whole country. The study also experienced time and fund constraints. It is maternal mental health research, and we faced difficulties conceptualizing and exploring it with a different experience of women in the community. These difficulties have been overcome after repeated meetings and consultations with experts in the area from the government health system of Bangladesh.

## Conclusion

The study documented some supply-side barriers to providing maternal mental health services in community-level health facilities such as CC, Union Health and Family Welfare Centre, and Union Sub-Centre. As found, community-level health providers do not have the knowledge and skills set to provide mental health support during this time, nor do they have proper guidelines and training regarding this. Despite having the positive significance of mental health during the perinatal period, this service is not yet available in community-level public health facilities in Bangladesh. From the demand-side perspective, the study also explored that the participants were not aware of getting service points and treatment, including medication for maternal mental disorders. They did not know who could provide the treatment and its impact on the mother and child in their future. This study documented the healthcare-seeking behavior and community healthcare service providers’ knowledge (guideline and training) gap regarding psycho-social support maternal mental health during the perinatal period of women in rural Bangladesh. Therefore, we emphasized integrating maternal mental healthcare support within the existing health system and promoting community mobilization for improving maternal mental health in rural settings of Bangladesh. Besides, the findings or evidence may also help the policymakers and program implementers to formulate appropriate policies addressing the community-level peoples’ knowledge and service gaps identified in this study. Furthermore, well-designed epidemiological and clinical research is needed to generate evidence to improve the mental health of perinatal women services in the community in Bangladesh. In addition, a feasibility study could be done to know how a psychosocial support intervention can be effective for perinatal women and their baby’s cognitive development.

## Data Availability

All data used in this study is readily available by request through the corresponding author. These are qualitative transcripts majorly.
